# Tropical range extending herbivorous fishes gain foraging benefits by shoaling with native temperate species

**DOI:** 10.1038/s41598-025-19136-x

**Published:** 2025-10-08

**Authors:** Mario Minguito-Frutos, Xavier Buñuel, Candela Marco-Méndez, Neus Sanmartí, Grigorios Skouradakis, Jordi Boada, Jordi F. Pagès, Teresa Alcoverro, Rohan Arthur

**Affiliations:** 1 Centre d’Estudis Avançats de Blanes (CEAB-CSIC), Carrer d’Accés a la cala Sant Francesc 14, 17300 Blanes, Girona, Spain; 2 Facultat de Biologia, Departament de Biologia Evolutiva, Ecologia i Ciències Ambientals, Universitat de Barcelona, Avinguda Diagonal 643, 08028 Barcelona, Spain; 3 Hellenic Centre for Marine Research (HCMR), Institute of Marine Biology, Biotechnology and Aquaculture (IMBBC), Heraklion, Crete, Greece; 4 Biology Department, University of Crete, 70013 Heraklion Crete, Greece; 5 Laboratoire d’océanographie de Villefranche, Sorbonne Université, Paris, France; 6 Nature Conservation Foundation, Amritha 1311, 12th Cross, Vijayanagara 1st Stage, Mysore, 570017 India

**Keywords:** Range-extension, Mixed-species foraging, Rabbitfish, Species interactions, Tropicalization, Herbivory, Biogeography, Climate-change ecology, Ecosystem ecology, Invasive species

## Abstract

Tropicalization creates novel species assemblages in temperate ecosystems as range-extending species move. The sociality of range-extending species could facilitate their establishment, particularly if they associate with natives. The eastern Mediterranean Sea has witnessed widespread collapses of vegetated ecosystems since the arrival of the tropical rabbitfishes *Siganus rivulatus* and *Siganus luridus*. We explored whether mixed-species shoaling influenced the fish foraging activity of the novel herbivore assemblage. We recorded 250 shoals, 30% composed exclusively of native species (i.e., *Sarpa salpa* and *Sparisoma cretense*), 43% of rabbitfish, and 27% of species from both origins. *S. rivulatus* was the most gregarious species, establishing positive shoaling associations with *S. luridus* and *S. salpa*, whereas *S. cretense* was the least sociable. Foraging differed between native and range-extending species. Compared to native species in mixed-species shoals, range-extending species increased their bite rates with shoal size, both in mono- and multi-specific groups, indicating that they boosted their foraging efficiency (i.e., increased bites per minute) when foraging in large groups, regardless of which species they shoaled with. Native species, in contrast, only increased their bite rates with shoal size while foraging in mono-specific groups. Thus, our study shows that, unlike natives, range-extending species may gain foraging benefits from facilitative associations in mixed-species shoals. This gregarious behaviour could help explain the disproportionate herbivory pressures range-extending species impose on tropicalized ecosystems.

**Supplementary Information:**

The online version contains supplementary material available at 10.1038/s41598-025-19136-x.

## Introduction

Between a changing climate and market-driven removal of (bio)geographical barriers, species are experiencing an unprecedented shift in their historical ranges^1,2^. An upshot of this rapid global reshuffling is that species with no shared evolutionary history are being increasingly brought into contact with each other^3^. The resulting assemblages are entirely novel in their interactions, often causing larger impacts on natural communities than changing environmental conditions alone^4^. These processes are of particular concern in the ocean, where there are fewer movement barriers than on land; as temperatures rise, marine species are fast spreading into once inhospitable environments^5^, leading to the tropicalization of temperate seas around the world^6–8^. How native and range-extending species interact in these evolutionarily novel species encounters can shape the pace and impact of this tropicalisation^3,9^. It is likely that, originating from more diverse and structurally complex habitats, tropical range-extending species have a wider behavioural repertoire than native species, helping them occupy vacant ecological niches in temperate ecosystems with fewer biotic interactions^10–12^. In addition, range-extending species with similar functional niches to those found in temperate environments could also benefit from a preadaptation to the functioning of these recipient ecosystems^13^.

Range-extending species with gregarious social traits may be inherently better able to establish successfully in new locations^11,14,15^. By associating with native species, they could learn considerably about the distribution and palatability of local resources, either through cultural transmission or imitation^16^. Foraging in mixed-species shoals is an interaction that can be mutually beneficial for all participating species, as it may reduce overall predation risk and vigilance requirements, enhance the probability of locating, capturing and collectively defending scarce resources, and increase swimming efficiency, among other potential advantages^17,18^. However, it is important to distinguish mere co-occurrence from mixed-species foraging^19^. While patterns of spatial co-occurrence can suggest ecological compatibility or shared habitat preferences, they do not necessarily imply active behavioural interactions. Mixed-species foraging, by contrast, entails coordinated or tolerated proximate foraging between heterospecifics, often involving behavioural adjustments, facilitation, or risk mitigation strategies that go beyond chance encounters^20^. It is critical to determine whether these benefits accrue equally for all participants in mixed-species shoals^18^. Certain species could potentially be more conspicuous to predators^17,21^, or when resources are scarce, species could be forced to compete for both food and space with more experienced or more efficient shoal participants^17,22^. Hence, the ability to navigate these trade-offs will vary with the species involved (see the findings of ^15^ and ^22^). What seems clear though, is that generalist strategies and plastic life history traits, in relation to both shoaling associations and foraging/feeding behaviours, may facilitate the expansion of range-extending species at their novel distribution edges^9,11,12,23^.

How native and range-extending species interact in novel assemblages can have major impacts on the structuring and functioning of the temperate ecosystems they inhabit^8^. The impacts of this wave of tropicalization can be intense due to the spread of tropical herbivores^6^, which often impose a strong top-down control on plant-dominated ecosystems^24,25^. Herbivores can be transformative drivers of marine vegetation abundance, and in extreme cases can lead to regime shifts or functional extinctions in marine vegetated systems^26,27^. In fact, tropical range-expanding species have been implicated in the spread of barrens in temperate macroalgal communities around the world, leading to a widespread decline in the structure and functioning of temperate reefs^28–32^.

With the opening of the Suez Canal in 1869, the Mediterranean Sea has witnessed a record number of incursions from the Red Sea and the Indian Ocean, with about 800-1000 Lessepsian species documented in its waters^33^. This invasion, together with an unprecedented rise in the seawater temperature, is accelerating the rate of successful establishment of range-extending species^33–35^. However, few range-extending species have been as successful as the two herbivorous rabbitfish, *Siganus rivulatus* (Forsskål 1775) and *Siganus luridus* (Rüppell 1828). First recorded in the eastern Mediterranean in Israel in 1924^36^, their arrival coincides with the loss of dense communities of canopy-forming macroalgae in favour of ecosystems dominated by thin turfs or bare rock^28,30,37^. Their success in Mediterranean rocky reefs has been attributed to several factors apart from warming seas^37,38^. These factors include the significant ecophysiological and phenological plasticity of rabbitfish species^39,40^, the virtual absence of large predators^41,42^, and the relative absence of herbivorous competitors^43^. The Mediterranean has only two exclusively herbivorous fish species, the shoal-forming *Sarpa salpa* (Linnaeus 1758) and the relatively solitary *Sparisoma cretense* (Linnaeus 1758)^44^. Where these range-extending rabbitfish are present in the eastern Mediterranean, they are often observed shoaling with native herbivores (see e.g.,^45^).

This study investigates if co-occurring native and range-extending herbivore species form positive associations with each other, and if these associations help them obtain foraging benefits in mixed-species shoals. We then explore if mixed-species shoaling can be a mechanism contributing to the success of range-extending rabbitfish, resulting in the high herbivory pressure Mediterranean rocky reefs currently experience^37^. Specifically, we hypothesize that tropical range-extending species, such as rabbitfish, may positively associate with native Mediterranean species and form mixed-species shoals as they do with conspecifics^46^, to obtain foraging benefits in novel tropicalized environments. By engaging in this associative foraging strategy, we expect an increase in shoal sizes and individual foraging efficiency with potential consequences for both range-extending and native herbivorous fishes. To address these questions, we evaluated (i) the frequency and size of mixed-species shoals as a function of novel shoaling configurations (i.e., based on species origin) where these four herbivores co-occur (ii) the strength of pair-wise associations between native and range-expanding species, and (iii) how the foraging activity of native and range-extending fishes was shaped by the type (mono- and multi-specific shoals) and size of shoals formed by these species. To test objectives (i) and (ii) we evaluated group composition (based on species origin) and shoal sizes formed by native and range-extending members; while for objective (iii) we tracked the foraging activity of individual fishes belonging to independent shoals of different sizes and types.

## Results

### Novel shoaling configuration

**Fig. 1:**
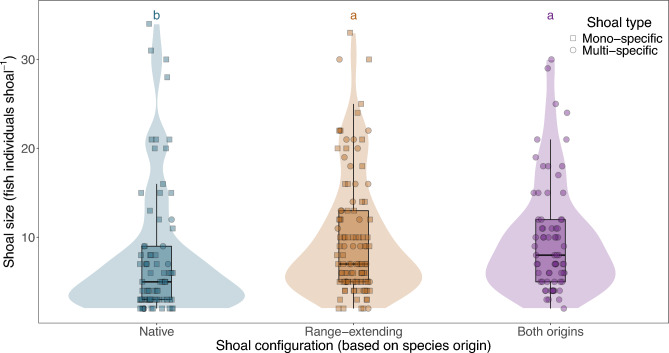
Relationship between shoal configuration and size of herbivorous fish shoals in Cretan rocky reefs. Shoal configurations, either in mono- or multi-specific groups, refer to shoals formed by native Mediterranean species (i.e., *S. salpa* and *S. cretense*), range-extending rabbitfish (i.e., *S. luridus* and *S. rivulatus*), and species of both origins. Results of Tukey test are shown with letters.

We encountered a total of 250 shoals of fish herbivore species across the seven locations studied; 30% of our observations were composed only of native species, 43% consisted exclusively of range-extending species and 27% of species from both origins (Fig. 1). Native species were rarely observed forming mixed-species shoals with each other, occurring in fewer than 3% of our observations. In contrast, range-extending rabbitfishes formed such associations significantly more often, in 14% of observations. This difference was statistically significant (χ^2^ = 12.879, df = 1, P < 0.001), indicating that rabbitfishes are more prone to forming mixed-species shoals. The likelihood of native species forming such associations was approximately five times lower (odds ratio = 0.19, 95% CI: 0.03–0.72, P = 0.009; Fisher’s Exact Test). Moreover, shoals consisting solely of native Mediterranean species (either in mono- or multi-specific groups) were smaller compared to shoals of range-extending species or shoals formed by native and tropical species (P < 0.001; Fig. 1; Supplementary Table S1).

### Species association strength

*S. rivulatus* was the most gregarious of all species (see Table 1), establishing positive associations (i.e., associations that were more likely than chance alone) with the range-extending *S. luridus* and the native *S. salpa*, and relatively neutral associations with the native *S. cretense*. In contrast, the rest of the herbivorous fish assemblage showed weak association values, with the native species *S. salpa* and *S. cretense* tending to avoid each other, as well as the range-extending *S. luridus*.

**Table 1:** Pairwise association strengths based on the co-occurrence matrix of shoals observed in the field. Positive values denote a higher degree of observed co-occurrences (i.e., association) than expected by chance, while negative values point to avoidance.

	*Sparisoma cretense*	*Siganus luridus*	*Siganus rivulatus*
*Siganus luridus*	-0.75		
*Siganus rivulatus*	-0.10	1.52	
*Sarpa salpa*	-0.93	-0.84	1.05

### Fish foraging activity

**Fig. 2:**
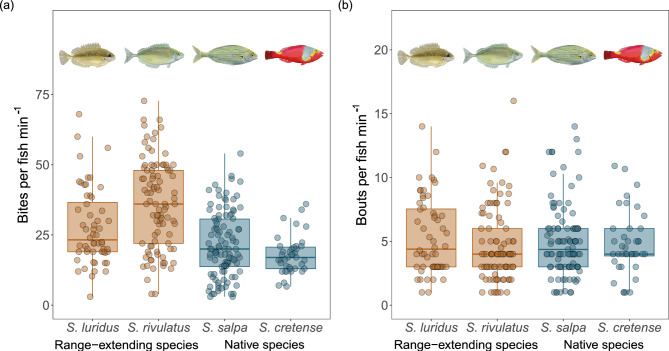
Field observations of fish foraging activity: (**a**) bite and (**b**) bout rates, as a function of species origin (i.e., range-extending and native species). Circles represent bite and bout rates per fish individual measured for each herbivorous species.

Field observations indicated that range-extending species exhibited higher average bite rates (mean + se = 32.9 ± 1.27; see Fig. 2a) than native species (mean + se = 20.5 ± 0.89). Average bout rates, however, remained more stable between both groups (mean + se = 4.96 ± 0.24 and 4.94 ± 0.23; for range-extending and native species, respectively; see Fig. 2b).

**Fig. 3:**
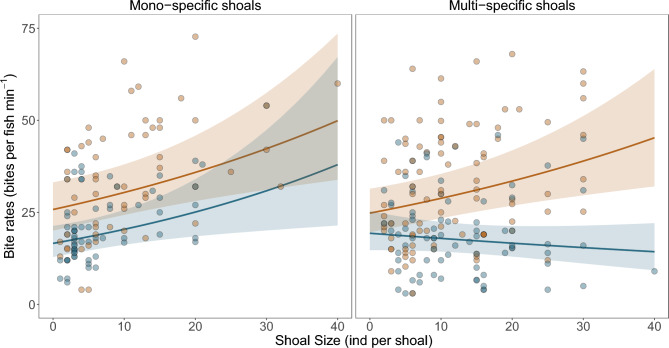
Bite rates predicted from our GLMM (Tweedie error structure), including a three-way interaction among species origin, shoal type, and shoal size. Lines and shaded ribbons show model-predicted smooths with 95% confidence intervals. Circles represent bite rates collected in the field for range-extending (brown) and native species (blue).

Range-extending species increased their bite rates in mixed-species shoals, unlike native species (Fig. 3). Our model revealed a significant three-way interaction term among species origin, shoal type and shoal size, influencing fish foraging activity (P = 0.036; Table S2). Specifically, range-extending species increased their bite rates with shoal size, both in mono- (β = 0.017; Table S3) as well as multi-specific shoals (β = 0.015; Table S3). Native species in contrast, also increased their bite rates with shoal size, but only in mono-specific shoals (β = 0.021; Table S3) and not in multi-specific shoals (β = -0.008; Table S3). Tukey-adjusted pairwise comparisons detected that range-extending species exhibited higher bite rates both in mono-specific (β = 0.024, P = 0.025; Table S4) and multi-specific shoals (β = 0.023, P = 0.032; Table S4) compared to native species in multi-specific shoals. Bites rates of native species were also greater when they shoaled in mono-specific groups than when they shoal with others (β = 0.028, P = 0.036; Table S4). There were no statistical differences in the slopes of range-extending fishes when shoaling in mono- and multi-specific shoals (β = 0.0015, P = 0.997; Table S4). These results indicate that, at the smallest group sizes (~3 individuals), range-extending species have a significantly higher baseline foraging activity than native species. While in mono-specific shoals the bite rates of native species were 65.1% of the rate of range-extending species (ratio = 0.651, P = 0.004), in multi-specific shoals, they foraged at 72.9% of the rate of range-extending species (ratio = 0.729, P = 0.034). These results show that, at these small group sizes, bite rates of range-extending species were 53% and 37% higher than those of native species in mono- and multi-specific shoals, respectively. In addition, bite rates decreased significantly with fish individual length (β = -0.020, P < 0.001; Table S2). Finally, contrasting with bite rates, our models for bout rates did not show significant interaction terms. Bout rates were explained only by shoal type, with fishes in mono-specific shoals exhibiting an overall 11% higher bout rate compared to those in multi-specific shoals (ratio = 1.11, Tukey-adjusted P = 0.039).

## Discussion

The novel assemblages of tropicalized seas force unfamiliar species to interact. The outcomes of these evolutionarily novel interactions could influence the success of range-extending species in temperate environments worldwide, with attendant consequences for their native communities and ecosystem functioning^6,8^. Our results show that gregarious range-extending herbivorous fishes may benefit from facilitative associations with other species in tropicalizing areas, by foraging collectively in mixed-species shoals. Multi-specific shoals with species from both biogeographic origins (i.e., Mediterranean and Red Sea) were relatively common in Crete, whereas native species rarely formed mixed-species shoals among them (less than 3% of our observations). Range-extending rabbitfishes exhibited a greater ability to shoal with other fish compared to the native *S. salpa* and *S. cretense*. By engaging in this mixed-species strategy, rabbitfish boost their effective shoal sizes, and thereby increase their feeding activity. In contrast, although native species also benefited from increasing shoal sizes, they did so only in mono-specific shoals. Altogether, our results show that the foraging benefits of mixed-species shoaling may vary asymmetrically with species origin (i.e., native Mediterranean vs. tropical), and favour tropical rabbitfishes, contributing to their growing success in their occupied waters.

Co-occurrence on its own does not imply associative foraging^19,20^. Mixed-species shoaling is behaviourally complex, requiring accurate information-sharing between species to coordinate the formation of shoals, determine their movement patterns, identify feeding areas, and respond effectively to perceived threats^17,18^. Results from pair-wise associations showed that the range-extending species, *Siganus rivulatus* was the most gregarious in the assemblage, forming positive associations with most herbivores (except for *S. cretense*), while others, associated only weakly or even avoided each other. In general, rabbitfishes tended to associate more and form larger shoals when shoaling with others than native Mediterranean herbivorous fishes. As evident from our observations (see Fig. 1), mixed-species foraging was not, to our knowledge, a recorded behavioural strategy for the only two native Mediterranean herbivorous fish species before the arrival of these tropical rabbitfish^44^. The presence of mixed-species shoals in which specifically *S. salpa* forage with these rabbitfish species where they co-occur has only recently been observed (see e.g.,^45,46^).

One potential reason why rabbitfish are so successful in associating with others in the native assemblage may be highly linked to their morphological similarity with the ubiquitous Mediterranean species *S. salpa*. While at a first glance this morphological similarity may impose competitive costs for newcomers, an analysis of body shapes of invading species in the Mediterranean found morphological novelty as a good indicator of invasive success^47^. In the reasoning of the authors, the relative success of rabbitfish in the Mediterranean, as with other species, has been linked to the limited presence of native morphological analogs. What our observations suggest, in contrast, is that rabbitfish could offset any potential competitive effects with *S. salpa* with the benefits of mixed-species shoaling. Because these species are well matched in shape, size, colour, swimming style, and other life history features, their similarity could be actually advantageous, enhancing predator confusion and helping them override potential “oddity effects” that could accrue when shoaling in groups^21^. For instance, when individuals within mixed-species shoals differ in size, colour or behaviour, they tend to experience increased attack rates (see^21^ and references therein); whereas phenotypic similarity might lead to higher protective mimicry, conferring benefits in terms of predation avoidance^18^. This, along with other factors affecting vigilance or diet partitioning, may help explain why morphological similarity is often an important determinant of mixed-species shoaling^18^. The degree of similarity in body shape could also contribute to the lower sociality of *S. cretense* with tropical rabbitfish. The parrotfish *S. cretense* was the most morphologically dissimilar species in the assemblage we studied, and occupied a slightly separate niche, with a beak-like jaw modified to scrape coralline algae and turfs^48^. While other morphologically similar invasive parrotfish species like *Scarus ghobban* have been reported in the Mediterranean, their abundances are low^47^; at least part of the reason they have not been able to thrive may be their inability to find sufficiently gregarious behavioural counterparts they could shoal with^11,13^.

Tropical rabbitfish species seem to have the experiential competence required to forage in mixed-species groups, likely mediated by their historical origins in ecosystems with higher richness and functional diversity of herbivorous fishes^6,8,10^. Indeed, in their native Red Sea ecosystems, they represent less than two percent of the total abundance of coral reef herbivores^38,49^. In contrast, the shoal-forming bream *S. salpa*, which is known for performing complex cooperative strategies in large mono-specific groups^50^, may be at a disadvantage, ecologically unused to sharing its niche with other herbivores^43,44^. Having evolved in a more functionally diverse environment, rabbitfish may be better behaviourally equipped to interact with unfamiliar species in their adoptive environments^8,10^.

Social species can benefit from group-living strategies and facilitative associations with others they encounter by foraging collectively in mixed-species groups^17,18^. Our study revealed that the feeding activity of native and range-extending species, which we measured as bite rates, scaled with group size when shoaling in mono-specific groups. However, only range-extending rabbitfish species foraging in multi-specific groups increased their feeding activity with shoal size. Although mixed-species foraging is known to help species bulk up shoal sizes or gain social cohesion while reaping the benefits of feeding in larger groups^11,12^, native species, including the shoal-forming *S. salpa*, clearly could not benefit in this way in mixed-species shoals. Unlike rabbitfishes, they were unable to improve their foraging efficiency, despite increasing numbers in multi-specific shoals. The question that emerges is why *S. salpa*, unlike the other native herbivore *S. cretense*, still show a positive association as derived from our analysis on association strength (i.e., not only co-occurrence) with *S. rivulatus*. Indeed, although we did not evaluate why *S. salpa* continues to associate with *S. rivulatus*, it may be likely due to non-foraging benefits of mixed-species shoaling, including increased joint vigilance or predator confusion, among other factors^18^. Alternatively, given the high densities of *S. rivulatus* across Cretan waters (see Section S1 in Supplementary information), *S. salpa* may have little choice and may be forced to share its environment with the rabbitfish. Our results suggest that the interaction is an asymmetric association, with *S. rivulatus* likely seeking out *S. salpa* shoals, which the latter merely endures because the costs of avoidance (e.g., relinquishing feeding grounds) may be considerably greater than shoaling together. It is unclear how stable this coexistence is however, and with time, it could lead to a reduction in *S. salpa* abundance, as has been observed in other tropicalized environments of the eastern Mediterranean Sea^28,30,43^.

What is apparent is that although range-extending species may be unfamiliar with resources and predators in their new environments, those exhibiting more generalist behavioural strategies can benefit from previously unoccupied functional niches^9,12,23^. In contrast, native temperate herbivores, less diverse and specialized than their tropical counterparts, may be limited in their range of ecological functions as biotic interactors^8,30,51^. In fact, it has been proposed that both herbivores *S. luridus* and *S. rivulatus*, along with other invasive species from different functional groups, are rapidly occupying vacant ecological niches within the Mediterranean Sea (i.e., compared to the Red Sea), leading to higher establishment rates and species abundances^52^. This unprecedented success of tropical rabbitfishes, which extends beyond the Mediterranean Sea, has been linked to specific traits including their highly plastic ecophysiology that allows them to settle in increasingly hospitable environments, and their generalist foraging and behavioural strategies^30,39,40^. Such behavioural plasticity, common in rabbitfish and other successful range-extending species, may help explain the asymmetry in shoaling interactions and differing foraging benefits obtained from mixed-species shoaling between temperate and tropical range-extending species^11,53^. These reasons may indicate an important role of behavioural traits in increasing feeding benefits, and facilitating the pre-adaptation and occupation of vacant ecological niches suggested for Mediterranean and other temperate marine systems^13,52^.

As an intrinsically observational study, these results are not without their limitations that prevent strong causal inferences. The relatively short observational window, imposed by the high fission–fusion dynamics of mixed-species shoals, may have limited our ability to capture competitive interactions, including subtle or transient aggressive behaviours, which could influence foraging activity and potentially reduce the fitness of native species. Also, although we did not record strong behavioural responses to observers, and allowed focal animals to acclimate to our presence, we cannot completely discount systematic differences in sensitivity to human presence between native and range-extending species, which could influence our inferences. More robust inferences would require complementary experimental approaches in more controlled settings, which would help establish specific baseline feeding activities in the absence of conspecifics or heterospecifics, and clarify the extent of behavioural plasticity in response to social environments. Our results may also be influenced by unmeasured ecological variables such as resource availability and differences in dietary specialisation between species of both origins, which may have influenced the observed foraging patterns. Understanding long-term fitness consequences of mixed-species shoaling—particularly for native species—is crucial to assess how associative interactions could influence species persistence and ecosystem functioning under continued tropicalization. Finally, the geographic scope of our study was restricted to a single region, limiting the generalizability of our findings. Broader studies across other parts of the tropicalized Mediterranean, as well as other tropicalizing systems including those in freshwater and terrestrial domains, are needed to confirm the patterns we observed.

## Conclusion

How much range-extending herbivorous species succeed and whether they persist and dominate in the novel assemblages of the tropicalized ocean will largely depend on species-specific traits, ecosystem composition, and the ecological context of each tropicalized environment^8,47^. Among other crucial factors including sea water temperature and organismal physiology^30,37^, the success of range-extending species appears to have much to do with generalist social behaviours, which help tropical fishes improve their feeding strategies in temperate environments^9,12,23^. In the tropicalized rocky reefs of the eastern Mediterranean Sea, rabbitfish species appear to show a greater overall ability to forage in mixed-species shoals compared to native herbivores. This behavioural generalism could help explain the overwhelming herbivory pressures experienced by macrophyte communities in this transitional area since the arrival of these voracious herbivores^28,30^. The functional consequences of these novel herbivore interactions are quickly redefining normality in the world’s most invaded sea; this is part of a more global phenomenon, where the spread of tropical species is drastically influencing herbivory dynamics across temperate waters^6,51^. This new normality in temperate environments is seriously jeopardizing ecosystem functioning and services provided by marine vegetated habitats and could have severe socioeconomic repercussions for these regions^3,8^.

## Material and methods

### Ethics statement

The observational protocol was submitted to the ethics committee of the institutional authority 'Institut de Ciències del Mar (ICM)' (the research institute in charge of animal welfare in our region), which did not require a special permit. Since this study is based on visual censuses conducted in unprotected waters, observing the natural behaviour of four species of teleost fishes that are neither endangered nor protected and, given that there is no extractive sampling or manipulation of the animal models, possible interactions with the welfare of animal models were discarded. As a result of that, this scientific survey was not subject to any further approval, regulation, or licensing committee, based on animal welfare regulations imposed by the Spanish Ministry of Science and Spanish National Research Council. Additionally, the methodology and its description comply with ARRIVE regulations and guidelines (https://arriveguidelines.org).

### Study area and design

Our study was conducted in the island of Crete (Greece), in the eastern Mediterranean basin, where two native species, the herbivorous bream, *S. salpa* and the parrotfish, *S. cretense* have co-occurred with the two range-extending rabbitfish, *S. rivulatus* and *S. luridus* for at least the last few decades^54,55^. Our study was designed (i) to describe the composition of herbivore shoals in this tropicalizing transition zone, (ii) to evaluate pairwise association strengths between native and range-extending species, and (iii) to assess if shoaling with other species influences foraging activity of native and range-extending species. For this, we conducted two types of underwater visual censuses. In the first, we sampled independent shoals to characterize shoal configurations and species association strength. In the second, we conducted behavioural observations on individual fish within independent shoals to characterize foraging activities based on species origin (see two subsections below). Surveys were conducted around noon (between 11:00 am and 2:00 pm) to minimize the highly variable diurnal foraging activity of these species^55^. We selected seven locations (Agia Pelagia, Agios Ioannis, Vathi, Krassas, Elounda, Psaromoura and Hersonissos) where herbivorous fish densities were high and all four species co-occur (see Fig. S1, Fig. S2, and Table S5 in Section S1 in Supplementary information for the map of the locations and data on species abundance). All surveys were carried out while snorkelling in shallow rocky reefs (depth range 0 – 6 m). For further details on the study area and the abundance of herbivorous fish species, see Section S1 in Supplementary information.

### Determining shoaling configurations and species association strength

To quantify the occurrence of different shoaling configurations and the strength of species associations, we recorded the composition of independent shoals encountered in free swims (n = 250 shoals as research units) at the seven sampling locations (see Fig. S1). Three observers conducted these surveys after inter-calibrating measurements to minimize variability. We sampled shoals opportunistically (the first observed regardless of size or composition) and each shoal was followed at a distance of a few meters until the fish were accustomed to the observer’s presence – resuming normal foraging behaviour and not showing escape responses. We recorded the species participating in every shoal and their abundance to determine the configuration of the encountered shoals and the strength of their association.

### Quantifying fish foraging activity

To determine how the origin of the species influenced their foraging activity, we measured species-specific foraging activity (i.e., bite and bout rates) of the four focal herbivorous fish individuals in mono- or multi-specific groups (of all different combinations), along a gradient of shoal sizes. We recorded bite rates (bites min^-1^) by visually counting the number of times one individual within each shoal took distinct bites to the substrate within the observation period, and bout rates (bouts min^-1^) as the number of discrete feeding events, separated by noticeable displacement or foraging between bites. In total, we quantified the foraging activity of 294 independent individuals within independent shoals (i.e., research units) across all four species (*S. salpa*, n = 105; *S. luridus*, n = 56; *S. rivulatus*, n = 93; *S. cretense*, n = 40) in six of the seven shallow-water locations (we did not sample fish foraging activity in Agios Ioannis, see Fig. S1).

We followed fish individuals and observed their foraging activity for up to two minutes^45^. Observations included a 30-second acclimation period for each sampled individual. Given the high rate of fissions and fusions in these mixed-species shoals, the average duration of our observations rarely exceeded one minute. We adjusted the time elapsed of our observations of mono-specific shoals to match that of multi-specific shoals. The observation was aborted if the fish showed evident responses to the observer or significantly changed its activity mode (e.g., from feeding to swimming). Observations less than or equal to 20 seconds were excluded from the analysis. To minimize ontogenic effects^50^, we collected the size of the targeted individual from each shoal, and restricted our observations to fish larger than 10 cm body length (Fig. S3). We measured the following parameters for every observation: i) species identity of the focal individual; ii) its body length; iii) the size of the shoal; iv) the type of the shoal (mono- or multi-specific); and v) the most abundant species present within the shoal.

### Data analysis

#### Shoaling configurations: species composition and size of the shoal

We quantified the frequencies of distinct shoaling configurations across the seven studied locations, categorizing shoals based on species origin (i.e., native-only, range-extending-only, or shoals with species from both origins; n = 250 shoals as research units). In addition, we used generalized linear mixed models (GLMMs) to test how the response variable ‘Shoal size’ varied with ‘Shoal configuration’ (fixed factor, three levels: native only, range-extending only, native and range-extending). Accordingly, we fitted a GLMM with log-normal error structure after visually and statistically evaluating the fit of four likely distributions (gamma, log-normal, Weibull, and negative binomial) using the *fitdistrplus* R package^56^ (see Supplementary Table S6). Initially, we included ‘Shoal type’ (two levels: mono- and multi-specific shoals) as fixed factor, and its interaction with ‘Shoal configuration’. ‘Location’ and ‘Observer’ were set as random factors. We then followed a stepwise model selection procedure, starting with a model of the structure ‘Shoal size’ ~ ‘Shoal configuration’ x ‘Shoal type’ + (1 | ‘Location’) + (1 | ‘Observer’); selecting the final model based on AIC and log-likelihood ratio tests (LRTs)^57^. Both random effects contributed minimally to the model (i.e., variance ± SD: ‘Location’ 0.016 ± 0.13; ‘Observer’ < 0.001 ± < 0.001). Based on these values, we first excluded the ‘Observer’ random factor due to matrix singularity issues indicating that it explained negligible variance. Then, after considering LRTs and AIC, and finding no substantial improvement in model fit, we also dropped the random factor ‘Location’. Our final (G)LM model (i.e., gaussian distribution) included only ‘Shoal configuration’ as fixed factor, since the fixed factor ‘Shoal type’ was also excluded based on AIC and LRTs to improve model parsimony. Moreover, we conducted a Fisher’s Exact Test to evaluate if frequencies of multi-specific shoals between categories of distinct origin (i.e., native-only or range-extending-only) were statistically significant.

#### Species association strength

Our analysis of association strength was motivated by the need to detect non-random, functionally relevant patterns of interaction between native and range-extending species, to distinguish co-occurrence from an active association that could influence foraging efficiency, vigilance, or habitat use^20^. Pairwise species association strengths were evaluated based on species co-occurrence in shoals. All independent observations of shoals across all locations were compiled in a presence-absence matrix with species in rows and shoals in columns (n = 250 shoals, see above). We used a randomization procedure using the EcoSimR R package^58^ to generate 1000 null matrices from our observed matrix, maintaining row totals (species richness remains unchanged) and shuffling column totals (see^59^). This allowed us to test observed co-occurrences against expectations by chance alone. We then calculated an index of pairwise species association strengths (*α*) using the probabilistic formula:

$$\alpha =\frac{O-\mu }{\sigma }$$

Where *O* is the number of species co-occurrences in our observed dataset, *μ* is the average of co-occurrences of that species pair in 1000 null matrices and *σ* is the standard deviation of the number of co-occurrences across the 1000 null matrices. *α* is a dimensionless index, where positive values indicate a stronger than expected co-occurrence (i.e., association), negative values indicate avoidance and zero indicates neutral or no interaction.

#### Fish foraging activity

We used two GLMMs with a Tweedie error structure to assess how individual fish foraging activities—specifically, bite rates and bout rates (bites and bouts per fish min^-1^)— relative to one focal individual within 294 independent shoals varied with group composition (see Table S2). For each response variable, we fitted a model that included all possible interactions among the fixed predictor variables: ‘Species origin’ (two levels: native and range-extending), ‘Shoal type’ (two levels: mono- and multi-specific shoals), and ‘Shoal size’. ‘Individual length’ was also included as a fixed factor. ‘Location’, ‘Observer’ and ‘Species identity’ were set as random factors to account for potential non-independence within these groups. The full model structures were: ‘Bite rates’ and/or ‘Bout rates’ ~ ‘Species origin’ x ‘Shoal size’ x ‘Shoal type’ + ‘Fish length’ + (1| ‘Location’) + (1| ‘Observer’) + (1| ‘Species identity’). We applied a stepwise model selection procedure (i.e., using AIC and LRTs^57^; see data analysis section on shoaling configuration above) on both models. For bite rates, the full model, with all factors included, was the most informative; for bout rates, the minimum adequate model retained only the fixed factor ‘Shoal type’ and the random factors.

All analyses were performed using the R language for statistical computing^60^. We removed a few outliers (n = 6) of shoals greater than 50 individuals from our datasets since it was difficult to get accurate measures of shoal size. GLMMs with a Tweedie error structure were fitted using the *glmmTMB*^61^ R package. We visually and statistically checked the assumptions of all fitted models using the *DHARMa*^62^ R package. Specifically, we assessed the normality, dispersion and presence of outliers, as well as the expected distribution of residuals via quantile regression (i.e., using the *DHARMA* functions^62^, *testUniformity()*, *testDispersion()*, *testOutliers()* and *testQuantiles()*, respectively). Model visualization was conducted using the estimates and 95% confidence intervals predicted by the *ggpredict()* function from the *ggeffects*^63^ R package. Where applicable, we performed post-hoc Tukey-adjusted pairwise comparisons via the *pairs()* function from the *emmeans* R package^64^.

## Supplementary Information

Supplementary Information 1.

## Data Availability

Data and R code that support the findings of this study are archived in Zenodo: 10.5281/zenodo.16901181
