# Supplementary material for: Tropical range extending herbivorous fishes gain foraging benefits by shoaling with native temperate species
Source: Sci Rep. 2025 Oct 8;15:35137. doi: 10.1038/s41598-025-19136-x (PMC12508093; doi:10.1038/s41598-025-19136-x)
Supplement: Supplementary file 1 — Supplementary Information 1. [file 41598_2025_19136_MOESM1_ESM.pdf]

Supplementary Information

Tropical range extending herbivorous fishes gain foraging benefits by shoaling with native temperate species

Scientific Reports

Mario Minguito-Frutos[1*], mminguito@ceab.csic.es; 0000-0003-3792-6782.

Xavier Buñuel[1], xavib06@gmail.com; 0000-0001-9064-3912.

Candela Marco-Méndez[1], c.marco@ceab.csic.es; 0000-0003-4278-5363.

Neus Sanmartí[2], neussanmarti@ub.edu; 0000-0001-8503-1006

Grigorios Skouradakis[3,4], gskouradakis@hcmr.gr; 0000-0002-8482-460X

Jordi Boada[5], jordi.boada@imev-mer.fr; 0000-0002-3815-625X.

Jordi F. Pagès[1], jpages@ceab.csic.es; 0000-0001-9346-8312.

Teresa Alcoverro[1,6], teresa@ceab.csic.es; 0000-0002-3910-9594.

Rohan Arthur[6,1], rohan@ncf-india.org; 0000-0003-4267-9720

[1] Centre d'Estudis Avançats de Blanes (CEAB-CSIC), Carrer d'Accés a la cala Sant Francesc 14, 17300 Blanes, Girona, Spain.

[2] Departament de Biologia Evolutiva, Ecologia i Ciències Ambientals, Facultat de Biologia, Universitat de Barcelona, Avinguda Diagonal 643, 08028, Barcelona, Barcelona, Spain.

[3] Hellenic Centre for Marine Research (HCMR), Institute of Marine Biology, Biotechnology and Aquaculture (IMBBC), Heraklion, Crete, Greece.

[4] Biology Department, University of Crete, 70013 Heraklion Crete, Greece

[5] Laboratoire d'océanographie de Villefranche, Sorbonne Université.

[6] Nature Conservation Foundation, Amritha 1311, 12th Cross, Vijayanagara 1st Stage, Mysore, 570017, India.

Corresponding author*: mminguito@ceab.csic.es

Keywords: *range-extension, mixed-species foraging, species interactions, tropicalization, herbivory, rabbitfish.*

Table S1. Type III F-tests of fixed effects from the minimum adequate model (i.e., after stepwise model selection) GLM model with log-normal distribution of errors, which explains shoal size (i.e., number of fish per shoal) as a function of shoal configuration.

Predictors	*Sum Sq*	*Df*	*F-value*	*P - value*
Intercept	957.59	1	2093.67	**< 0.001*****
Shoal configuration	7.77	2	8.49	**< 0.001*****
Residuals	112.97	255		

Table S2. Type III Wald chi-squared tests of fixed effects from GLMMs models (Tweedie distribution) explaining fish foraging activity (i.e., bites and bouts per fish min^{-1}). The full model for bite rates was the most informative, retaining all interactions among species origin (i.e., native and range-extending species), shoal type (i.e., mono- and multi-specific shoals), and shoal size; and the effect of the predictor variable individual length. The minimum adequate model for bout rates only included the fixed factor shoal type.

Predictors	*Chisq*	*Df*	*P - value*
**Bites per minute**			
Intercept	59.36	1	**< 0.001*** **
Species origin	6.33	1	**0.012***
Shoal type	0.43	1	0.512
Shoal size	11.02	1	**<0.001*****
Individual length	11.52	1	**<0.001*****
Species origin * Shoal type	1.24	1	0.266
Species origin * Shoal size	2.07	1	0.151
Shoal type * Shoal size	5.50	1	**0.019***
Species origin * Shoal type * Shoal size	4.42	1	**0.036***
**Bouts per minute**			
Intercept	106.87	1	**<0.001*****
Shoal type	4.25	1	**0.039***

Table S3. Slope estimates from the GLMM model (Tweedie distribution) explaining bites rates (bites per fish min^{-1}) as a function of shoals size, across the different combinations of species origin and shoal type. Confidence intervals (asymptotic 95% CI) are provided.

Species Origin	*Shoal type*	*Estimate*	*SE*	*Df*	*95% CI*
Native	Mono-specific	0.021	0.008	Inf	0.0047 - 0.0367
Native	Multi-specific	-0.008	0.007	Inf	-0.0207 - 0.0056
Range-extending	Mono-specific	0.017	0.006	Inf	0.0057 - 0.0273
Range-extending	Multi-specific	0.015	0.005	Inf	0.0053 - 0.0248

Table S4. Tukey-adjusted pairwise comparisons of the estimated slopes of shoal size on bite rates (bites per fish min^{-1}), across the different combinations of species origin and shoal type. Estimates represent differences in slopes between modelled group combinations. Statistically significant comparisons are indicated in bold.

Contrasts	*Estimate*	*Std. Error*	*Df*	*z-ratio*	*P - value*
Range-extending mono-specific – Native multi-specific	0.024	0.009	Inf	2.82	**0.025***
Range-extending multi-specific – Native multi-specific	0.023	0.008	Inf	2.73	**0.032***
Range-extending mono-specific – Range-extending multi-specific	0.002	0.007	Inf	0.21	0.997
Native mono-specific – Native multi-specific	0.028	0.011	Inf	2.69	**0.036***
Native mono-specific – Range-extending mono-specific	0.004	0.010	Inf	0.43	0.973
Native mono-specific – Range-extending multi-specific	0.006	0.010	Inf	0.60	0.932

Section S1: Abundance of herbivorous fishes in the study site

MATERIALS AND METHODS

Study area

The study was conducted in the island of Crete (Greece) in September 2021. Crete, in the eastern Mediterranean, has an herbivorous fish assemblage, where two native species, the herbivorous bream, *Sarpa salpa* and the parrotfish, *Sparisoma cretense* have co-occurred with the two range-extending rabbitfish, *Siganus rivulatus* and *S. luridus* for at least the last few decades[1,2]. We sampled four locations along the north coast (in the Cretan Sea): Agia Pelagia, Psaromoura, Hersonissos, Elounda; and three locations along the south coast (in the Libyan Sea): Agios Ioannis, Vathi and Krassas (see caption in Fig. S1). Most reefs were dominated by photophilic turf algae typical of early colonization stages, or with canopy-forming species of fucales, composed mostly of *Cystoseira spp*. (*sensu lato)*, close to the surface. At all sites, reefs were adjacent to (or mixed with) sandy areas, with patches of *Posidonia oceanica* and *Cymodocea nodosa* completing the mosaic of these highly variable coastal areas.

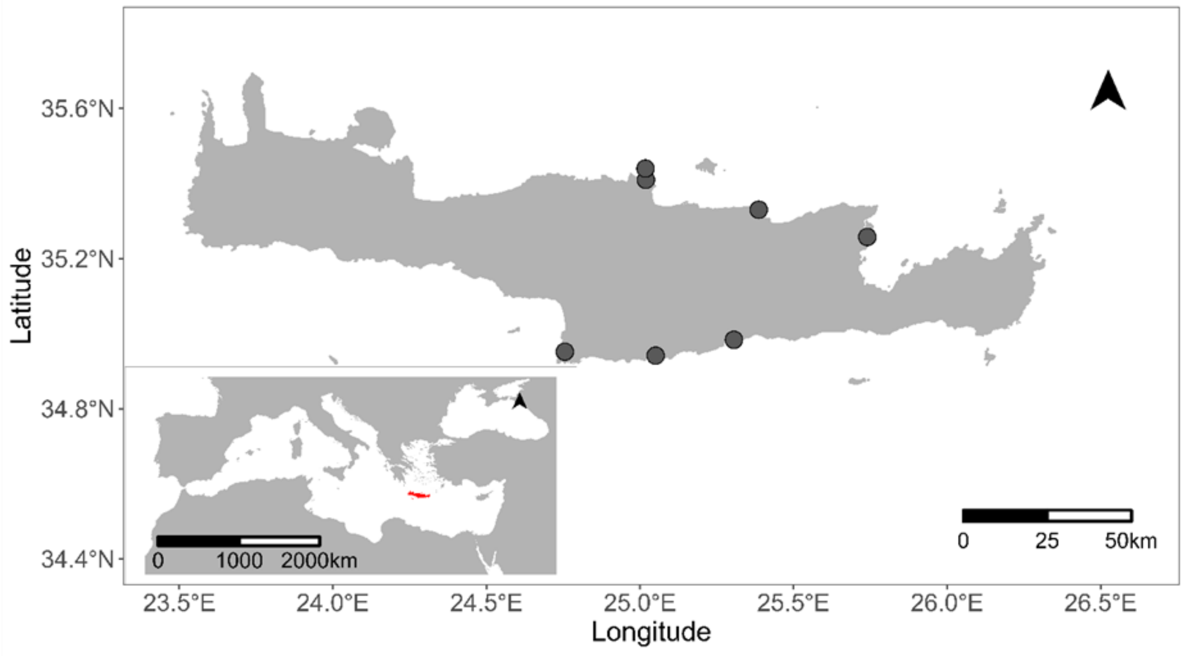

Fig. S1. Sampling locations on the north coast (Cretan Sea), Psaromoura, Agia Pelagia, Hersonissos and Elounda (from west to east, respectively); and on the south coast of Crete (Libyan Sea), Vathi, Agios Ioannis and Krassas (from west to east, respectively). In the lower left corner, Crete's location in the Mediterranean Sea is highlighted in red.

Characterizing abundances of herbivore shoals

We quantified the abundance of native and range-extending herbivorous fish species using visual transects of 50 m x 5 m (n = 3 per location) at six locations of Crete's shallow rocky reefs (Agia Pelagia, Psaromoura, Elounda, Agios Ioannis, Vathi and Krassas). Replicate transects were separated by at least 10 m[5]. Along the transect, the observer recorded the type (mono- and multi-specific) and size of each shoal (number of individuals), as well as the number of individuals of each species and their average individual sizes (cm).

Data Analysis

Abundances of herbivore shoals

We examined differences in fish abundance per species and shoal type with a linear mixed model (LMM). In this LMM, we set ‘Abundance’ (fish transect^{-1}) as the response variable, and ‘Species’ (four levels: *S. salpa*, *S. luridus*, *S. rivulatus* and *S. cretense*), and ‘Shoal Type’ (two levels: mono- and multi-specific shoals) as fixed predictor variables. ‘Location’ (six levels: Agia Pelagia, Psaromoura, Elounda, Agios Ioannis, Vathy and Krassas) was included as a random factor to control for site-level variance. All the assumptions of linear models were met after logarithmic transformation of the response variable. We evaluated model assumptions with visual and statistical examination of residuals using the R packages, *performance*[6] and DHARMa[7].

RESULTS

Abundances of herbivore shoals

Most species in the herbivore fish assemblage in Crete were found predominantly in shoals. Along the transects, individuals were encountered in shoals 89% of the time for *Sarpa salpa*, 75% of the time for *S. rivulatus*, and 60% of the time for *Siganus luridus*. Only the native parrotfish *S. cretense* was observed almost equally in shoals (49%) as alone. However, herbivore abundance did not vary between mono- and multi-specific shoals ($P = 0.479$), and there was no evidence of an interactive effect of shoal type and species ($P = 0.515$) (Table S5; Fig. S2). In contrast, herbivorous fish abundance varied strongly across species ($P < 0.001$) (Table S5; Fig. S2). *S. rivulatus* was by far the most abundant in Crete’s herbivorous fish assemblage (mean ± se = 33 ± 4.48 individuals per transect), with abundances nearly 3 times higher than the native *S. salpa* (mean ± se = 11.5 ± 3.48 individuals per transect), and eight times greater than its congener *S. luridus* (mean ± se = 4.10 ± 0.87). The abundance of the native *S. salpa* was significantly higher than *S. cretense,* which was the least abundant herbivore (mean ± se = 2.67 ± 0.51).

Table S5. Type III Wald chi-squared tests of fixed effects from LMMs explaining fish abundance (fish per transect^{-1}) as a function of species and shoal type. Location was set as a random factor.

**Predictors**	**Chisq**	**Df**	***P - value***
(Intercept)	27.16	1	< **0.001*****
Species	70.76	3	< **0.001*****
Shoal type	0.50	1	0.479
Species * Shoal type	2.29	3	0.515

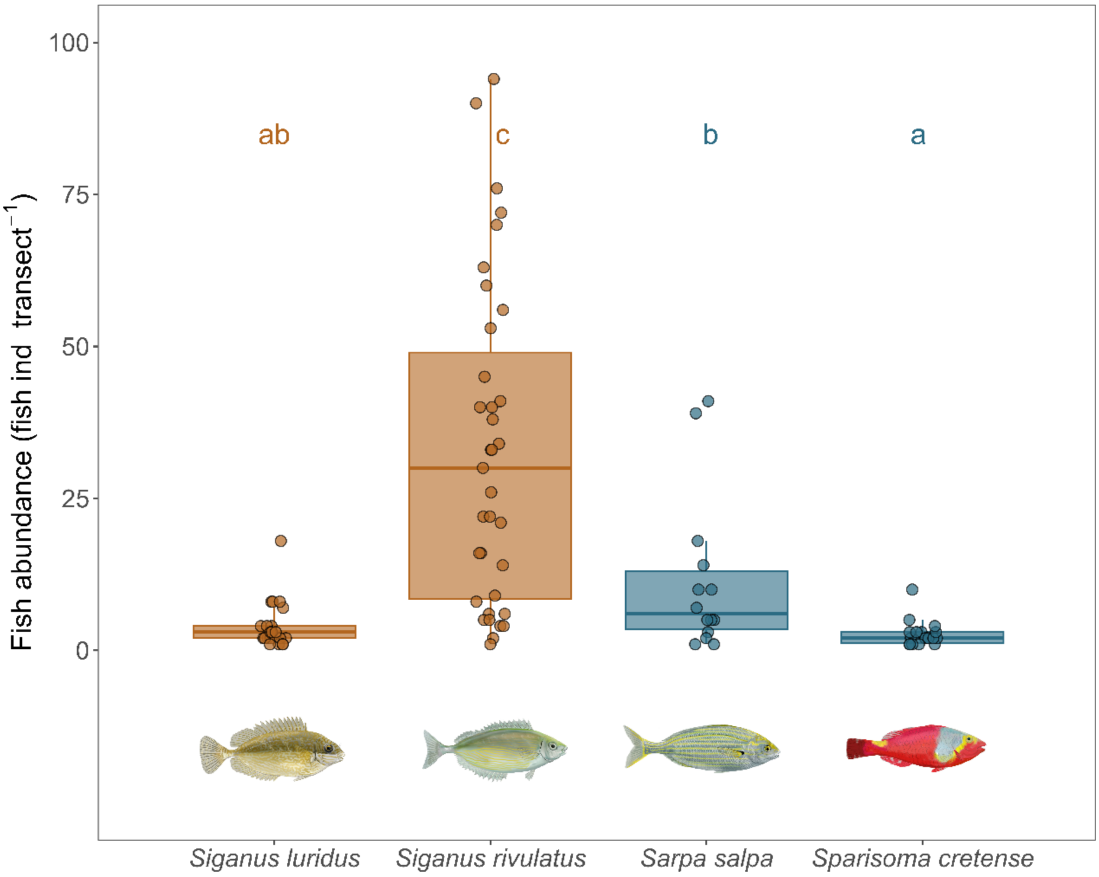

Fig. S2. Relationships between fish abundance (fish individuals transect^{-1}) and the predictor variable 'Species'. Circles represent field observations of the number of individuals measured of the four fish herbivorous species. Letters represent significant differences between species based on pairwise Tukey comparisons, using the Kenward-Roger approximation to estimate degrees of freedom.

Table S6. Goodness-of-fit statistics and criteria for selecting the distribution of the response variable ‘Shoal size’.

**Goodness-of-fit statistics for shoal size**				
	Gamma	*Log-normal*	*Weibull*	*Neg. Binomial*
Kolmogorov-Smirnov	0.1238	**0.0783**	0.1273	0.1056
Cramer-Von Mises	0.5689	0.2110	0.6956	0.4959
Anderson-Darling	3.3554	1.4684	4.2907	4.2437
**Goodness-of-fit criteria for shoal size**				
AIC	1595.306	1575.442	1611.792	1611.128
BIC	1602.412	1582.547	1618.898	1618.234

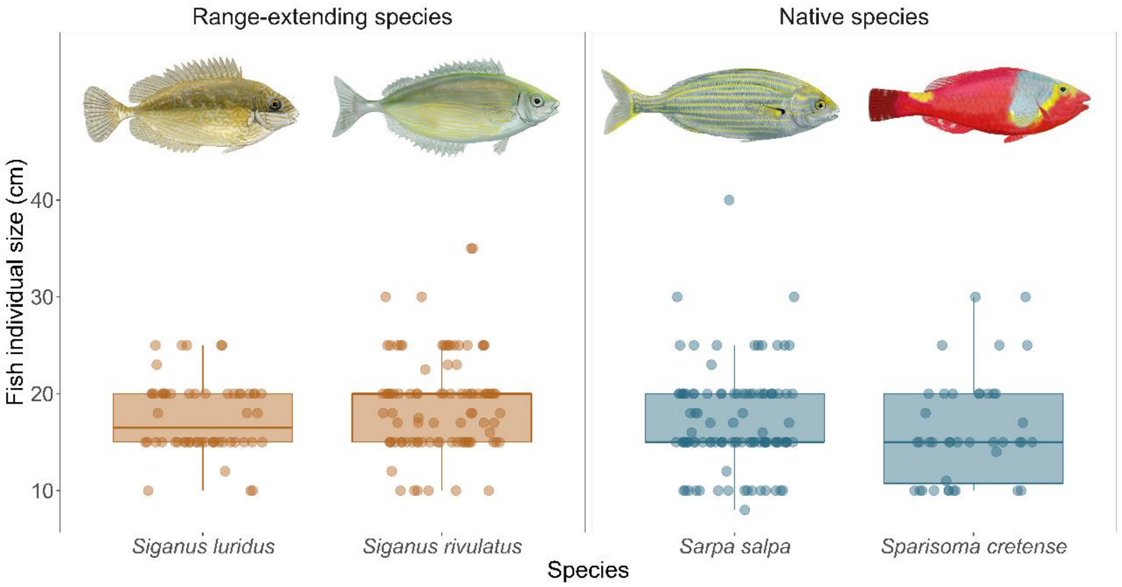

Fig. S3. Body size distribution of the 294 individual fish from the focal species included in the study, for which bite and bout rates were measured.

REFERENCES

1. Stergiou, K. I. Feeding habits of the Lessepsian migrant *Siganus luridus* in the eastern Mediterranean, its new environment. *J Fish Biology* **33**, 531–543 (1988).
2. Magneville, C. *et al.* Long-duration remote underwater videos reveal that grazing by fishes is highly variable through time and dominated by non-indigenous species. *Remote Sens Ecol Conserv* rse2.311 (2022) doi:10.1002/rse2.311.
3. Willis, T. J., Badalamenti, F. & Milazzo, M. Diel variability in counts of reef fishes and its implications for monitoring. *Journal of Experimental Marine Biology and Ecology* **331**, 108–120 (2006).
4. Myers, E. M. V., Harvey, E. S., Saunders, B. J. & Travers, M. J. Fine-scale patterns in the day, night and crepuscular composition of a temperate reef fish assemblage. *Mar Ecol* **37**, 668–678 (2016).
5. Schramm, K. D. *et al.* A comparison of stereo-BRUV, diver operated and remote stereo-video transects for assessing reef fish assemblages. *Journal of Experimental Marine Biology and Ecology* **524**, 151273 (2020).
6. Lüdecke, D., Ben-Shachar, M., Patil, I., Waggoner, P. & Makowski, D. performance: An R Package for Assessment, Comparison and Testing of Statistical Models. *JOSS* **6**, 3139 (2021).
7. Hartig, F. Dharma: residual diagnostics for hierarchical (multi-level/mixed) regression models (2022). – https://cran.r-project.org/web/packages/DHARMa/vignettes/DHARMa.html
